# Short-term efficacy of physical interventions for lateral epicondylitis: a network meta-analysis based on multidimensional evaluation of pain and function

**DOI:** 10.3389/fpain.2026.1855508

**Published:** 2026-06-26

**Authors:** Yu-Hong Song, Jin-Yan Lan, He-Hui Fu, Li-Xu Tang

**Affiliations:** Martial Arts Academy, Wuhan Sports University, Wuhan, Hubei, China

**Keywords:** dry needling, lateral epicondylitis, meta-analysis, percutaneous electrolysis, physical interventions

## Abstract

**Background:**

Lateral epicondylitis (LE) is a prevalent chronic tendinopathy that significantly impairs upper limb function and quality of life. This network meta-analysis (*N*MA) aims to evaluate and compare the short-term (≤3 months) efficacy of various micro-invasive and physical interventions for pain relief and functional restoration, providing an evidence-based framework for tailored clinical decision-making.

**Methods:**

A comprehensive search was performed in PubMed, Embase, the Cochrane Library, Web of Science, and CNKI for randomized controlled trials (RCTs) investigating physical interventions for LE. Primary outcomes included the Visual Analogue Scale (VAS), the Disabilities of the Arm, Shoulder and Hand (DASH) score, and the Patient-Rated Tennis Elbow Evaluation (PRTEE). Data synthesis, consistency testing, and Surface Under the Cumulative Ranking curve (SUCRA) calculations were performed using Stata 18.0. Two-dimensional cluster analysis was employed to concurrently assess the analgesic and functional benefits of the interventions.

**Results:**

Analysis of 38 RCTs revealed that percutaneous electrolysis (PE) was the most effective intervention for pain reduction (SUCRA = 98.5%), yielding a clinically pronounced effect size against baseline care (MD = −6.00, 95% CI: −9.75 to −2.25) and outperforming most other physical therapies. Dry needling (DN) demonstrated the highest ranking probability in functional improvement (DASH SUCRA = 79.7%; PRTEE SUCRA = 85.2%), and was the sole active treatment significantly superior to conservative care on the PRTEE scale (MD = −25.04, 95% CI: −48.29 to −1.79). In the cluster analysis, PE was localized to the “strong analgesia” quadrant, while DN was situated in the “strong functional recovery” quadrant. Other interventions, such as platelet-rich plasma and corticosteroid injections, exhibited only moderate effectiveness.

**Conclusion:**

The investigated interventions yield distinct therapeutic profiles for LE within an early temporal window. Percutaneous electrolysis shows the highest probability for rapid pain mitigation, whereas dry needling presents a favorable probability profile for restoring localized elbow function. However, given the high underlying network heterogeneity, the lack of statistical significance in DASH-related pairwise comparisons, and overlapping prediction intervals, these SUCRA-based rankings should be interpreted cautiously. We do not recommend formulating definitive long-term clinical recommendations based on these early temporal profiles; instead, clinical decisions should be tailored to the patient's predominant short-term symptoms—balancing immediate pain relief with early targeted functional recovery.

**Systematic Review Registration:**

https://www.crd.york.ac.uk/prospero, PROSPERO CRD420251234552.

## 1 Introduction

Lateral epicondylitis (LE), commonly referred to as “tennis elbow,” is a chronic tendinopathy characterized by degenerative changes in the extensor tendons, particularly involving the extensor carpi radialis brevis (ECRB) muscle (1). Epidemiological data indicate a prevalence rate of 1%–3% among the general adult population (2),with a notably higher incidence in individuals engaged in prolonged, repetitive wrist-intensive labor. Patients typically present with debilitating lateral elbow pain and diminished grip strength, which not only severely restricts activities of daily living but also imposes a substantial socioeconomic burden due to lost productivity (3). Currently, a wide array of conservative management strategies is available for LE. Physical interventions have emerged as first-line clinical preferences owing to their non-invasive nature and favorable safety profiles. Common modalities include dry needling (DN), extracorporeal shockwave therapy (ESWT), and the increasingly utilized percutaneous electrolysis (4–7). However, clinicians often face a lack of clear, evidence-based guidance when selecting among these diverse options. Although numerous randomized controlled trials (RCTs) have evaluated various therapies, most have focused on isolated outcome measures and lacked head-to-head comparisons between different active interventions. Consequently, clinical decision-making often remains empirical and somewhat arbitrary. Furthermore, previous meta-analyses have predominantly emphasized pain mitigation while overlooking the nuanced recovery of elbow joint function. Emerging evidence suggests that a reduction in pain intensity, as measured by the Visual Analogue Scale (VAS), does not consistently correlate with concurrent improvements in elbow-specific function (PRTEE score) or overall upper limb performance (DASH score). To address these discrepancies, a holistic evaluation integrating multidimensional indicators is imperative. Network meta-analysis (NMA) extends traditional meta-analytic techniques by facilitating the ranking of multiple interventions within a single evidence network through both direct and indirect comparisons.

The objective of this study is to perform a systematic and comprehensive evaluation of the short-term efficacy of various physical interventions in alleviating pain and enhancing multidimensional function in patients with LE. By employing NMA, SUCRA rankings, and two-dimensional cluster analysis, we aim to explore the equilibrium between analgesic effects and functional rehabilitation. This approach endeavors to provide clinicians with robust evidence to support the development of individualized, precision treatment protocols.

## 2 Methods

### 2.1 Search strategy

This study was conducted in strict accordance with the Preferred Reporting Items for Systematic Reviews and Meta-Analyses (PRISMA) guidelines. A comprehensive systematic search was performed across major electronic databases, including PubMed, Embase, the Cochrane Library, Web of Science, and CNKI, from their respective inceptions through December 2025. The search strategy employed a combination of Medical Subject Headings (MeSH) and relevant keywords, including “lateral epicondylitis,” “tennis elbow,” “physical therapy,” “percutaneous electrolysis,” “dry needling,” “acupuncture,” and “randomized controlled trial.”

**Figure 1:**
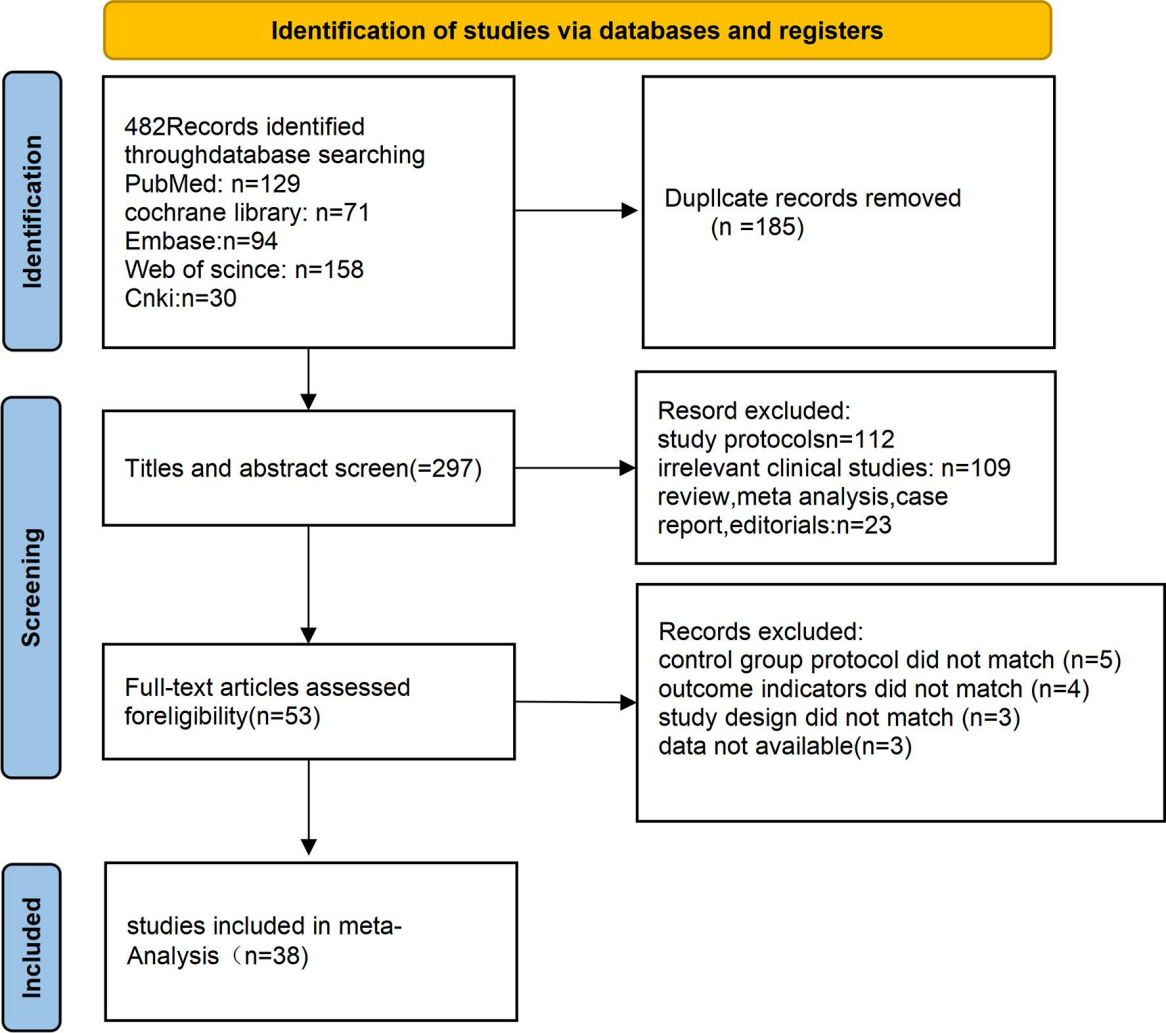
PRISMA flow diagram of search.

To ensure the literature search was exhaustive, we manually screened the reference lists of all retrieved studies and relevant systematic reviews to identify additional pertinent trials. Following the removal of duplicates, two reviewers (SYH and LJY) independently screened the titles and abstracts of the identified records. Subsequently, the full texts of potentially eligible articles were rigorously evaluated based on predefined inclusion and exclusion criteria. Any discrepancies during the selection process were resolved through consensus or, if necessary, by consultation with a third investigator (FHH), who also oversaw the data extraction process. The complete study selection process is summarized in the PRISMA flow diagram (Figure 1).

### 2.2 Inclusion and exclusion criteria

Studies were selected based on the following Inclusion Criteria:

Participants (P): Patients clinically diagnosed with lateral epicondylitis (LE), with no restrictions on age or gender.

Interventions (I): Targeted therapies including manual acupuncture, electroacupuncture, dry needling (with or without ultrasound guidance), percutaneous electrolysis (PE), Fu's subcutaneous needling, and minimally invasive injections [e.g., local corticosteroids or platelet-rich plasma (PRP)], either as monotherapies or in combination.

Comparisons (C): Control groups receiving conservative management, such as therapeutic exercise (eccentric/isometric), physical modalities (e.g., ultrasound, TENS), non-steroidal anti-inflammatory drugs (NSAIDs), or placebo/sham interventions (e.g., saline injections or sham acupuncture).

Outcomes (O): Studies must have reported at least one validated measure for pain or physical function, including the Visual Analogue Scale (VAS), the Patient-Rated Tennis Elbow Evaluation (PRTEE), or the Disabilities of the Arm, Shoulder and Hand (DASH) questionnaire.

Study Design (S): Only randomized controlled trials (RCTs) were included.

The Exclusion Criteria were defined as follows:Non-randomized designs, including cohort studies, case series, reviews, descriptive studies, and expert opinions. Studies involving patients with concomitant elbow pathologies (e.g., fractures, radial tunnel syndrome, or systemic inflammatory arthritis). Duplicate publications or studies utilizing overlapping datasets. Studies with incomplete data or those where outcomes could not be extracted or converted into mean ± standard deviation for meta-analysis.

### 2.3 Data extraction and quality assessment

Two researchers independently screened the literature and extracted data. Extracted information included: first author, publication year, sample size, intervention measures, follow-up duration, and outcome measures. The Cochrane Risk of Bias tool (RoB 2.0) was used to assess the quality of included studies, covering five domains: randomization process, deviation from the intended intervention, missing outcome data, outcome measurement, and selective reporting of results. To standardize clinical interpretability, follow-up durations across the included randomized controlled trials were rigorously classified based on established rehabilitation literature frameworks: short-term (defined as ≤3 months post-randomization), medium-term (>3 months to 6 months), and long-term (>6 months). Given that the primary therapeutic mechanisms of invasive micro-needling (such as percutaneous electrolysis and dry needling) focus on mitigating acute chemical/mechanical nociception and initiating early tissue remodeling, and that available raw clinical data in the included trials overwhelmingly clustered around early time points, this network meta-analysis specifically targeted and pooled short-term outcomes to ensure maximal data homogeneity and statistical power.

To maintain a mathematically stable network geometry and ensure sufficient statistical power for indirect loops, active physical interventions were clustered into predefined, broader therapeutic nodes based on their primary biophysical mechanisms rather than micro-technical variations. Specifically, within the “dry needling” node, although distinct clinical protocols exist—such as localized myofascial trigger point (MTrP) needling, deep intramuscular stimulation, and superficial static needling—they fundamentally share the core mechanical identity of inserting a solid filament needle into affected tissues without injectate to disrupt structural contractures, elicit a local twitch response, and stimulate regional hyperemic healing. Splitting these subtypes into isolated nodes would fragment the network structure, causing broken loops and an inflation of statistical imprecision.

To rigorously evaluate the selection of the reported result domain (reporting bias), the reviewers systematically searched major international and domestic trial registries (e.g., ClinicalTrials.gov, ChiCTR). Among the 38 included RCTs, only 1 trial provided a verifiable prospective registration record. While 33 trials provided institutional ethics committee approval numbers, their prospective statistical protocols were not publicly accessible in registration databases, and 5 trials lacked any reported approval information. To maintain a rigorous and conservative methodological standard, all 38 studies were scrutinized by cross-checking the methods section against the reported results within the text. However, due to the lack of independent, pre-published protocol verification, the remaining trials were conservatively graded as having “some concerns” or “high risk” in the selective reporting domain, which transparently accounts for the higher proportion of non-low-risk trials in our overall reporting bias assessment.

### 2.4 Data synthesis and statistical analysis

Network meta-analysis was performed within a frequentist framework using Stata 18.0 software. For continuous outcomes, including VAS, PRTEE, and DASH scores, the mean difference (MD) where appropriate—along with its corresponding 95% confidence interval (CI) was utilized as the pooled effect size. Initially, a network plot was constructed to visualize the evidence structure and the comparative relationships between interventions. In this visualization, the size of the nodes corresponds to the total sample size for each intervention, while the thickness of the connecting lines represents the number of studies included for each direct comparison. The core assumption of network meta-analysis, namely transitivity, was evaluated by assessing the comparability of clinical and methodological characteristics across the included trials. Two independent reviewers rigorously examined the baseline characteristics of participants (e.g., age, symptom duration, and baseline severity), intervention details (e.g., protocol frequency and dosage), follow-up periods, and allowed concomitant treatments to ensure that the populations were sufficiently similar across different treatment comparisons. To rigorously evaluate the impact of clinical and methodological diversity on the evidence network, quantitative heterogeneity was comprehensively assessed using a random-effects meta-regression model. Global residual variation due to heterogeneity was quantified via the residual *I*^2^ statistic and the between-study variance (*τ*²). Furthermore, the statistical consistency of the network was multi-dimensionally evaluated based on its topological configuration. For closed-loop structures (VAS and DASH networks), global inconsistency was tested using the design-inconsistency framework (via the network meta i package), where an *F*-test was applied to evaluate the joint hypotheses of all inconsistency parameters. Additionally, the statistical consistency of individual split nodes was rigorously evaluated using the local node-splitting method. For acyclic or radial network configurations lacking closed loops (PRTEE network), where independent indirect pathways do not mathematically exist, the consistency assumption was satisfied by structural design. A *P*-value > 0.05 was considered to indicate no significant inconsistency between direct and indirect evidence, justifying the use of a consistency model. The relative hierarchy of the interventions was established by calculating the Surface Under the Cumulative Ranking curve (SUCRA). Higher SUCRA values indicate a greater probability of an intervention being among the most effective. Furthermore, a two-dimensional cluster analysis was performed to concurrently evaluate the therapeutic benefits across both pain and functional dimensions. Finally, potential publication bias and small-study effects were assessed using a comparison-adjusted funnel plot.

### 2.5 Certainty of evidence assessment

To evaluate the certainty of evidence for each networked comparison, a formal assessment was conducted using the GRADE (Grading of Recommendations Assessment, Development and Evaluation) framework adapted for network meta-analyses (NMA), consistent with CINeMA methodologies. Evidence for primary outcomes (VAS, DASH, and PRTEE) was scrutinized across six structural domains: within-study bias, reporting bias, indirectness, imprecision, heterogeneity, and incoherence. The certainty of evidence for each unique therapeutic loop was subsequently categorized as high, moderate, low, or very low. A visual summary matrix displaying these semi-quantitative risk levels was generated to enhance the transparency of the evaluation.

## 3 Results

### 3.1 Characteristics of included studies

The initial literature search identified 54 potentially relevant records. Following the removal of duplicates and subsequent evaluation of titles, abstracts, and full texts, 38 randomized controlled trials (RCTs) were ultimately included in the network meta-analysis. The baseline characteristics of the included studies are summarized in Table 1.

**Table 1:** Characteristic of included studies.

Author, year	Sample size	Mean age (year)		Mean ± SD		Experimental group	Control group	Duration (weeks)	Outcomes
	(Experiments/control)	Treatment	Control	Treatment	Control				
Katrine Bostrøm 2019	13/11	51 (4)	47 (3)	4.00 (1.10)	4.41 (1.11)	Acupuncture	Exercise	4	NRS score
Gregor Stenhouse 2013	12/13	47.6 (6.12)	53.2 (9.87)	6.87 (2.15)	8.07 (1.19)	Dry Needling	ACP group	8	VAS score
Renee Keijsers MD 2024	53/57	51 ± 8	53 ± 8	6.9 ± 2	6.9 ± 2	Percutaneous	Dextrose	8	VAS score
						Perforation			
Lasse Linnanmaki MD 2020	40/39	46 ± 5	49 ± 8	5.7 ± 1.7	5.9 ± 1.8	Platelet-rich plasma	Saline	4	DASH score/VAS score
Elif Umay Altas, MD 2022	26/26	45.5 ± 7.1	47.0 ± 6.6	8.5 ± 1.2	8.3 ± 1.2	Dry Needling	Exercise	3	DASH score/VAS score/PRTEE score
Esat UYGUR 2018	49/52	47.5 ± 7.3	48.1 ± 10.3	60.9 ± 11.8	58.6 ± 5.1	Dry Needling	Corticosteroid	3	PRTEE
Blanca De-la-Cruz-Torres 2021	12/12	49.5 ± 9.5	49.4 ± 5.5	7.7 ± 0.9	7.6 ± 1.15	PNM	Waiting list for treatment	4	NRS score/PRTEE score
James Dunning 2024	73/70	42.0 ± 9.9	43.4 ± 11.0	42.0 ± 11.5	42.7 ± 15.6	Dry Needling	Multimodal physical therapy	4	PRTEE
Lin-Pu Ge 2022	34/34	46.2 (19–65)	44.2 (21–63)	6.8 (1.6)	6.4 (1.3)	Acupotomy	Corticosteroid injection	6	VAS score
Chung-Yuan Hsu 2016	19/16	45.89 ± 5.99	44.81 ± 7.30	6.5 ± 2.54	6.21 ± 1.26	Acupuncture	Manipulation	8	DASH score/VAS score
Xinjian Li 2014	40/40	19 ± 2	19 ± 1	6.5 ± 1.9	6.4 ± 1.6	Electroacupuncture + massage + blocking therapy;	Blocking therapy	4	VAS score
Fatma Gü lç in Ural, MD 2017	18/23	42.1 ± 8.6	46.9 ± 8.6	8.1–1.0	8.2–1.6	Acupuncture	Usual care	4	VAS score
Clara Wing-Yee Wong 2017	17/17	/	/	6.12 ± 2.09	5.47 ± 1.97	Acupuncture	ESWT extracorporeal	3	DASH score/VAS score
							shockwave therapy		
Dr. Sheikh Irfan Bashir 2020	24/24	36.95 years (24–56 yrs)	38.79 years (20–58 ）	7.54 (0.73)	7.54 (0.73)	Corticosteroid	Platelet rich plasma	1	VAS score
Usama Bin Saeed 1 2018	19/19	43 ± 12	47 ± 61	7.3 ± 2.1	7.2 ± 1.9	Steroid	Platelet rich plasma	6	VAS score
Madhuram Chowdry 2017	30/30	38.1 (9.3)	40.1 (8.15)	4.85 ± 1.09	4.60 ± 0.94	Patelet rich plasma	Steroid	2	DASH score/VAS score
Dr. Partha Pratim Das 2019	90/86	39.13	39.8	7.36 ± 0.891	6.98 ± 1.051	Platelet rich plasma	Steroid	2	DASH score/VAS score
VK Gautam 2015	15/15	/	/	7.1 ± 0.8	7.0 ± 0.8	Platelet rich plasma	Corticosteroid	2	DASH score/VAS score
Erdal Güngör 2021	24/24	46.08 ± 7.44	40.91 ± 7.70	8.16 ± 0.81	8.0 ± 0.83	Dry Needling	Corticosteroids	3	DASH score/VAS score
Taco Gosens 2011	49/51	47.3 6 ± 7.8	46.8 ± 8.5	6.62 ± 1.4	6.9 ± 1.59	Corticosteroid	Platelet-Rich Plasma	4	DASH score/VAS score
P. Kumar Gupta 2019	40/40	/	/	8.1 ± 0.85	4.45 ± 1.73	Platelet-Rich Plasma	Corticosteroid	6	DASH score/VAS score
Manjunath S Japatti 2020	20/20	/	/	6.97 ± 0.38	6.69 ± 0.48	Platelet-Rich Plasma	Steroid	4	DASH score/VAS score
Prashant Kamble 2023	32/32	/	/	7.75 ± 0.56	8.62 ± 0.49	Platelet-Rich Plasma	Steroid	3	DASH score/VAS score/PRTEE score
Mahmoud El Tayeb Nasser 2017	15/15	40.93 ± 8.46	41.67 ± 4.23	6.5 ± 1.67	6.8 ± 0.86	Platelet-Rich Plasma	Steroid	12	VAS score/PRTEE
Aziza Sayed Omar 2012	15/15	40.5 ± 15.5	37.5 ± 17.5	8.0 ± 1.4	8.6 ± 1.6	Platelet-Rich Plasma	Corticosteroid	6	DASH score/VAS score
Evandro Pereira Palacio 2016	20/20	46.6	46.2	45.7 ± 3.8	44.3 ± 4.4	Platelet-Rich Plasma	Corticosteroid	12	DASH score/PRTEE
KS Sandhu, 2020	25/25	44.2	42.6	7.08 (0.99)	7.54 (0.73)	Platelet-Rich Plasma	Corticosteroid	3	VAS score
Shahram Sayadi, MD 2023	15/15	47.67 ± 11	50.27 ± 12	9 ± 2	9 ± 0.25	Platelet-Rich Plasma	Corticosteroid	4	DASH score/VAS score
Ankit Varshney 2017	33/50	/	/	8.33 ± 1.08	2.45 ± 0.90	Platelet-Rich Plasma	Corticosteroid	4	VAS score
Jain P 2020	50/49	43.40 ± 8.38	45.32 ± 8.31	6.74 ± 0.37	6.8 ± 0.5	Dry Needling	Platelet-Rich Plasma	24	DASH score/VAS score
Hamid ullah Khan, 2018	50/50	/	/	7.1 ± 1.6	7.2 ± 1.8	Platelet-Rich Plasma	Corticosteroid	2	VAS score
Muhammad Usman Khalid 2016	50/50	39.38 ± 10.58	43.00 ± 8.04	7.38 ± 1.38	7.62 ± 1.42	Platelet-Rich Plasma	Corticosteroid	2	DASH score/VAS score
Abdul Khaliq 2015	51/51	34 ± 1	34 ± 10	6.7 ± 1.4	6.5 ± 1.2	Platelet-Rich Plasma	Corticosteroid	3	VAS score
Abdul Qadeer Khan Sadiq 2023	81/81	48.1 ± 8.2	47.4 ± 7.5	7.74 ± 1.06	7.69 ± 1.03	Platelet-Rich Plasma	Steroid	6	VAS score
Dr. Chandanpreet Kaur 2023	46/46	41.2 ± 8.14	39.72 ± 7.49	6.67 ± 1.30	6.48 ± 1.50	Platelet-Rich Plasma	Steroid	4	VAS score/PRTEE
LI Shengwu 2025	27/28	/	/	6.41 ± 0.93	6.36 ± 0.91	Dry Needling	Blocking therapy	2	VAS score
MA Xuejian 2024	31/30	36.6 ± 5.8	35.9 ± 6.1	6.19 ± 0.98	6.27 ± 1.01	Dry Needling	Corticosteroid	4	DASH score/NRS score
Liu Nai-cheng 2021	32/32	51.44 ± 6.59	52.26 ± 5.25	7.22 ± 0.61	7.06 ± 0.72	Platelet-Rich Plasma	Corticosteroid	4	VAS score

The included trials encompassed seven distinct categories of interventions, classified as follows:Group A: Conservative care including exercise therapy alone (e.g., eccentric/isometric training), basic physical agent therapy (e.g., ultrasound), oral or topical nonsteroidal anti-inflammatory drugs, and kinesiology tape. Group B: Placebo/sham controls included sham acupuncture, saline injection controls, and ineffective simulated physical therapy. Group C: Traditional acupuncture included manual acupuncture, electroacupuncture, and acupuncture based on Traditional Chinese Medicine meridian points. Group D: Dry needling included standard dry needling, ultrasound-guided dry needling, percutaneous micro-puncture, and Fu's subcutaneous needling (FSN). Group E: Defined and referred to as percutaneous electrolysis throughout this study. This modality involves the ultrasound-guided application of a galvanic current through a cathode needle into the damaged soft tissue (e.g., the common extensor tendon) to induce a localized biomodulatory and non-thermal electrolytic ablation. This modality is distinguished from non-invasive physical modalities, such as transcutaneous electrical nerve stimulation (TENS), which are categorized under the conventional physiotherapy or standard care node. Group F: Corticosteroid injections included local corticosteroid injections (e.g., dexamethasone). Group G: PRP includes platelet-rich plasma injections. All studies reported short-term efficacy measures post-treatment.

### 3.2 Methodological quality of the studies

**Figure 2:**
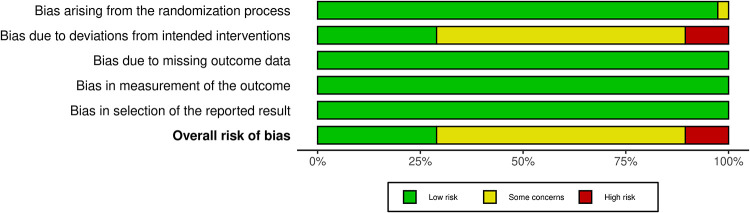
Bias risk map.

The methodological quality of the 38 included RCTs was evaluated using the Cochrane Risk of Bias 2 (RoB 2) tool (Figure 2). As illustrated in the domain-specific assessment, all 38 studies (100%) were rated as “low risk” for bias arising from missing outcome data and measurement of the outcome.

Regarding the randomization process, the majority of trials demonstrated a low-risk rating. Conversely, “deviations from intended interventions” was identified as the primary source of potential bias, with approximately 70% of the included studies flagged with “some concerns” or “high risk” in this domain. This primarily reflects the intrinsic methodological challenges of achieving double-blinding for both clinicians and participants in trials involving manual or invasive physical interventions (e.g., dry needling or percutaneous electrolysis).

Crucially, regarding the selection of the reported result domain, only the single prospectively registered trial was graded as “low risk,” whereas the remaining 37 trials were conservatively graded as having “some concerns” or “high risk” due to the absence of publicly accessible, pre-published statistical protocols. Consequently, the overall risk of bias assessment categorized 11 studies as “low risk,” 23 as having “some concerns,” and 4 as “high risk.”

### 3.3 Results of the network meta-analysis

**Figure 3:**
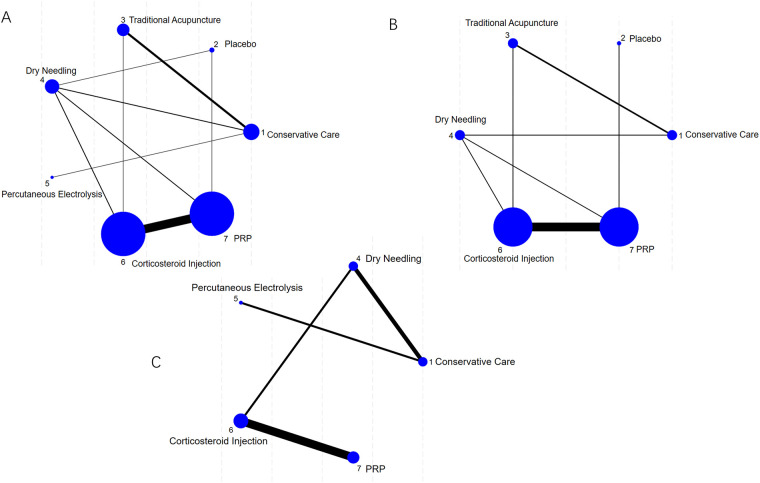
Network structure diagrams [**(A)** VAS, **(B)** DASH and **(C)** PRTEE].

As illustrated in Figure 3, evidence network diagrams were constructed for the three core clinical outcomes: VAS, DASH, and PRTEE. The VAS network formed the most comprehensive topology, integrating all seven therapeutic nodes (conservative care, sham/placebo, traditional acupuncture, dry needling, percutaneous electrolysis, corticosteroid injections, and PRP). In contrast, the functional networks were smaller, encompassing six interventions for the DASH scale (excluding percutaneous electrolysis) and five primary categories for the PRTEE scale.

Within the VAS and DASH networks, connectivity was well-distributed; the nodes representing corticosteroid and PRP injections occupied the largest geometric areas and engaged in the most frequent direct head-to-head comparisons, forming the core of the evidence layout. Conversely, the PRTEE network demonstrated a radial configuration centered on conservative treatment with limited local loops, reflecting a relatively sparse distribution of original trials.

For the closed-loop structures in the VAS and DASH networks, the local node-splitting method yielded no statistically significant differences between direct and indirect evidence (*P* > 0.05), indicating no significant inconsistency and justifying the application of the consistency framework. Percutaneous electrolysis exhibited a high connectivity value within the VAS metric, facilitating multi-pathway comparative loops. Collectively, these three networks constitute a complementary evidence system, providing a statistically sound foundation for evaluating the comparative benefits of invasive needling therapies.

### 3.4 Efficacy results of NMA

#### 3.4.1 Pain relief(VAS)

**Figure 4:**
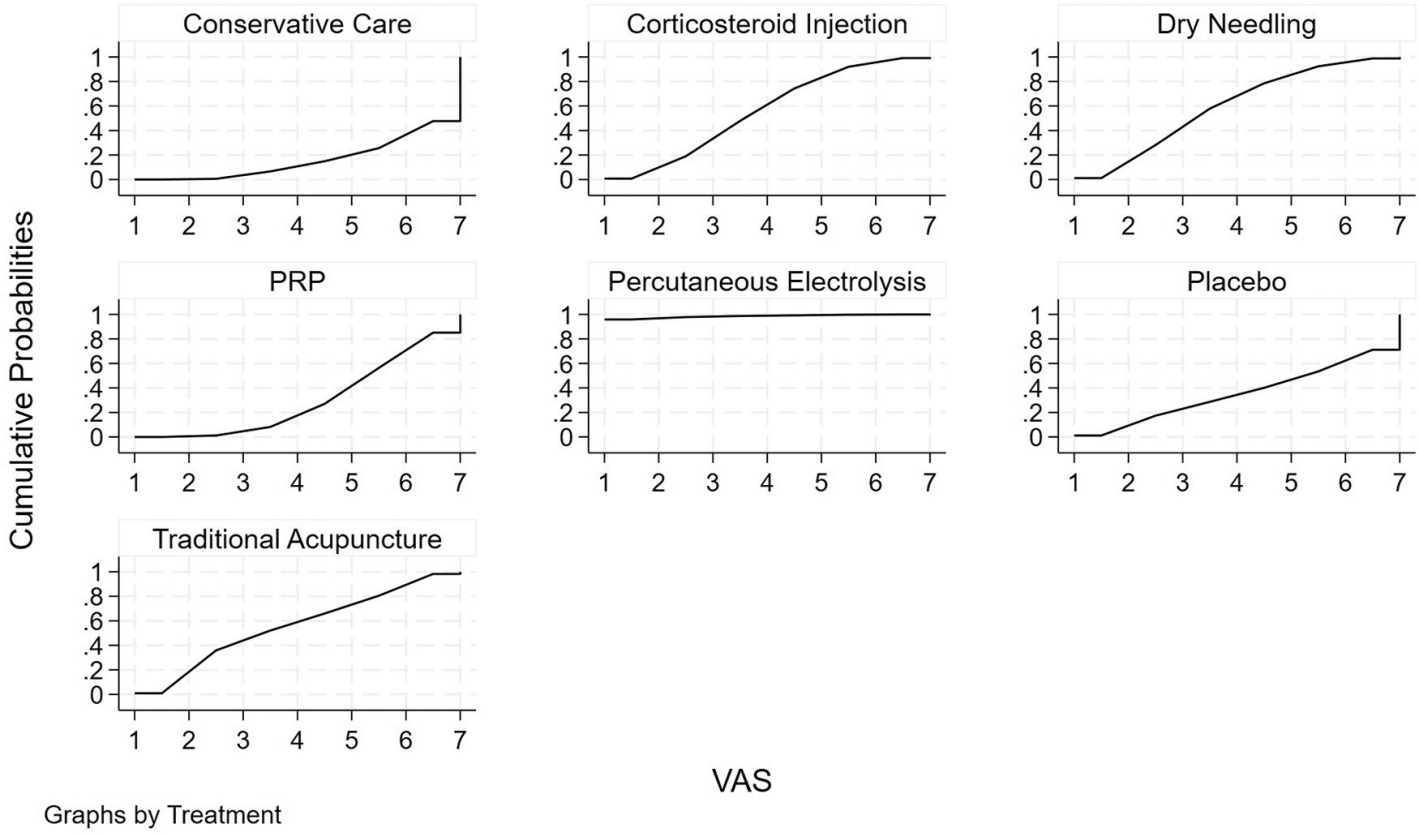
SUCRA plot.

**Figure 5:**
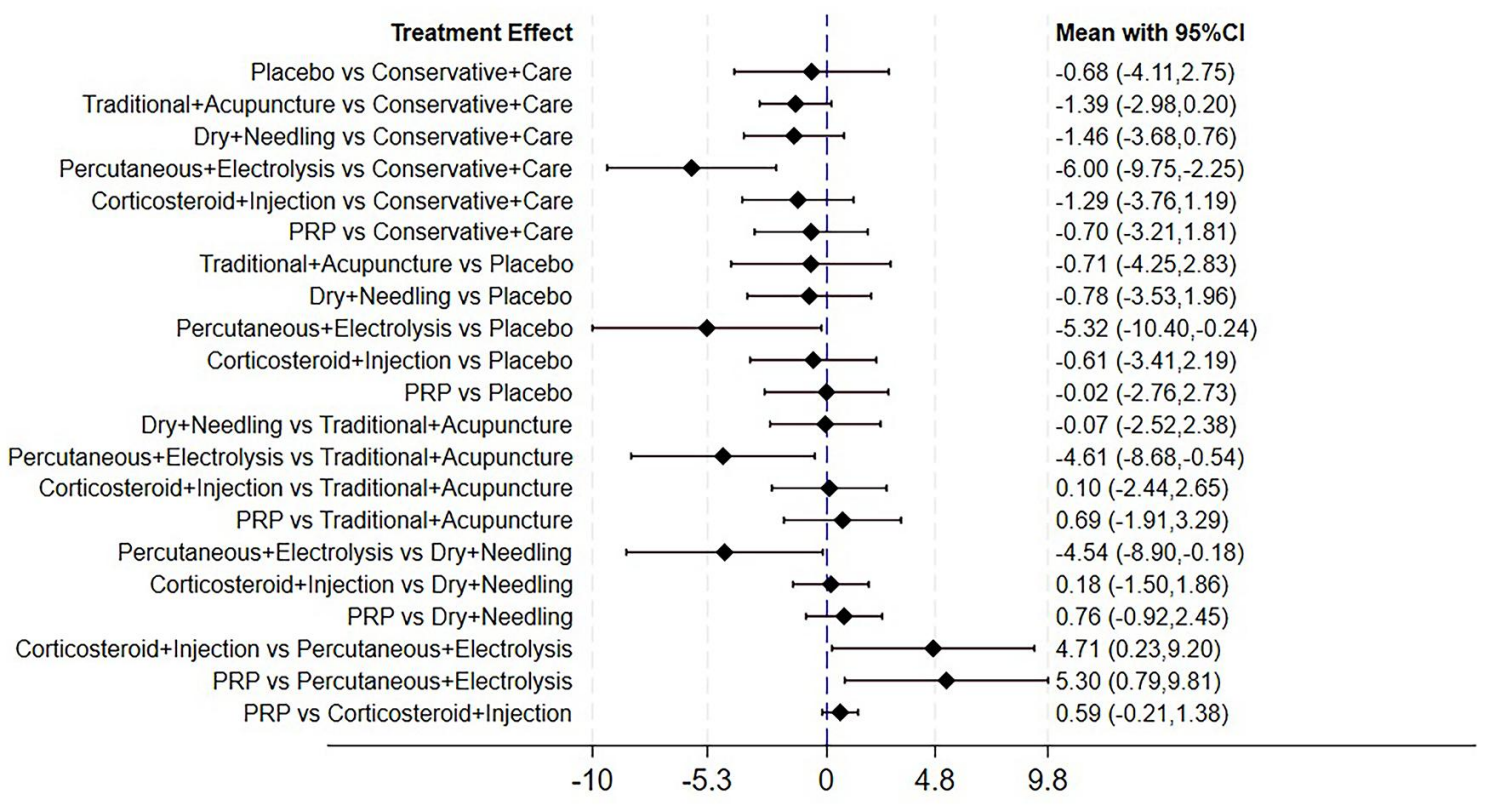
Forest plot of network meta-analysis for VAS comparing all interventions with conservative care.

The random-effects network meta-analysis revealed differences among physical interventions in mitigating short-term pain scores (Figures 4, 5). According to the SUCRA (Surface Under the Cumulative Ranking) analysis, percutaneous electrolysis demonstrated the highest probabilistic ranking, scoring a SUCRA value of 98.5% and a 95.9% probability of being the optimal intervention. This was followed hierarchically by dry needling (SUCRA = 59.5%), traditional acupuncture (SUCRA = 55.6%), and corticosteroid injections (SUCRA = 55.5%), whereas PRP (SUCRA = 29.7%) and conservative care (SUCRA = 15.9%) ranked the lowest.

In terms of direct pairwise comparisons (Table 2), percutaneous electrolysis significantly outperformed conservative care with a mean difference of −6.00 (95% CI: −9.75, −2.25). To evaluate the clinical relevance of this finding, the pooled MD was benchmarked against the established Minimal Clinically Important Difference (MCID) threshold for the VAS score in lateral epicondylitis (commonly anchored at 1.5–2.0 cm, or 15%–20% on a 0–10 scale). The point estimate of −6.00 exceeded the upper bound of the MCID threshold, indicating that the short-term pain relief provided by percutaneous electrolysis represents a clinically meaningful benefit to the patient within this evaluation window.

**Table 2:** League table for pairwise comparisons of pain intensity (VAS) among different physical interventions for lateral epicondylitis: a network meta-analysis.

**Conservative Care**	−0.68 (−4.11,2.75)	−1.39 (−2.98,0.20)	−1.46 (−3.68,0.76)	**−6.00** (−9.75,−2.25)*	−1.29 (−3.76,1.19)	−0.70 (−3.21,1.81)
0.68 (−2.75,4.11)	**Placebo**	−0.71 (−4.25,2.83)	−0.78 (−3.53,1.96)	**−5.32** (−10.40,−0.24)*	−0.61 (−3.41,2.19)	−0.02 (−2.76,2.73)
1.39 (−0.20,2.98)	0.71 (−2.83,4.25)	**Traditional Acupuncture**	−0.07 (−2.52,2.38)	**−4.61** (−8.68,−0.54)*	0.10 (−2.44,2.65)	0.69 (−1.91,3.29)
1.46 (−0.76,3.68)	0.78 (−1.96,3.53)	0.07 (−2.38,2.52)	**Dry Needling**	**−4.54** (−8.90,−0.18)*	0.18 (−1.50,1.86)	0.76 (−0.92,2.45)
**6.00** (2.25,9.75)*	**5.32** (0.24,10.40)*	**4.61** (0.54,8.68)*	**4.54** (0.18,8.90)*	**Percutaneous Electrolysis**	**4.71** (0.23,9.20)*	**5.30** (0.79,9.81)*
1.29 (−1.19,3.76)	0.61 (−2.19,3.41)	−0.10 (−2.65,2.44)	−0.18 (−1.86,1.50)	**−4.71** (−9.20,−0.23)*	**Corticosteroid Injection**	0.59 (−0.21,1.38)
0.70 (−1.81,3.21)	0.02 (−2.73,2.76)	−0.69 (−3.29,1.91)	−0.76 (−2.45,0.92)	**−5.30** (−9.81,−0.79)*	−0.59 (−1.38,0.21)	**PRP**

Data are presented as mean differences (MD) with 95% confidence intervals (CI). Comparisons should be read from column to row. Negative MD values indicate that the column intervention is superior to the row intervention (i.e., lower pain). Bold values with an asterisk (*) indicate statistically significant differences (95% CI does not cross 0).

A, Conservative Care; B, Placebo; C, Traditional Acupuncture; D, Dry Needling; E, Percutaneous Electrolysis; F, Corticosteroid; G, PRP.

The evidence suggests a probabilistic trend that percutaneous electrolysis holds the highest probability for short-term pain relief. However, while league tables show favorable point estimates, the wider confidence intervals and the lack of statistical significance in several other active pairwise comparisons suggest that these ranking hierarchies should be interpreted as probabilistic tendencies rather than clinical superiority.

#### 3.4.2 Functional recovery (DASH and PRTEE)

**Figure 6:**
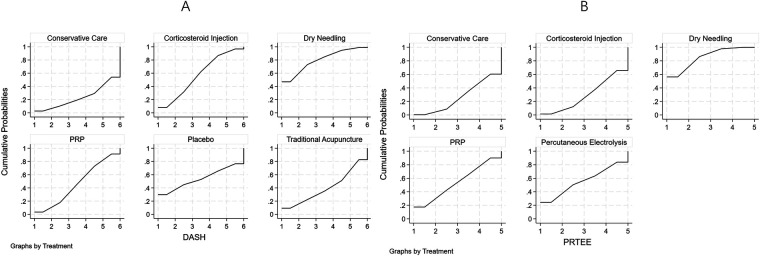
Surface under the cumulative ranking curve (SUCRA) plots of different interventions for the DASH score and PRTEE score [**(A)** DASH, **(B)** PRTEE].

**Figure 7:**
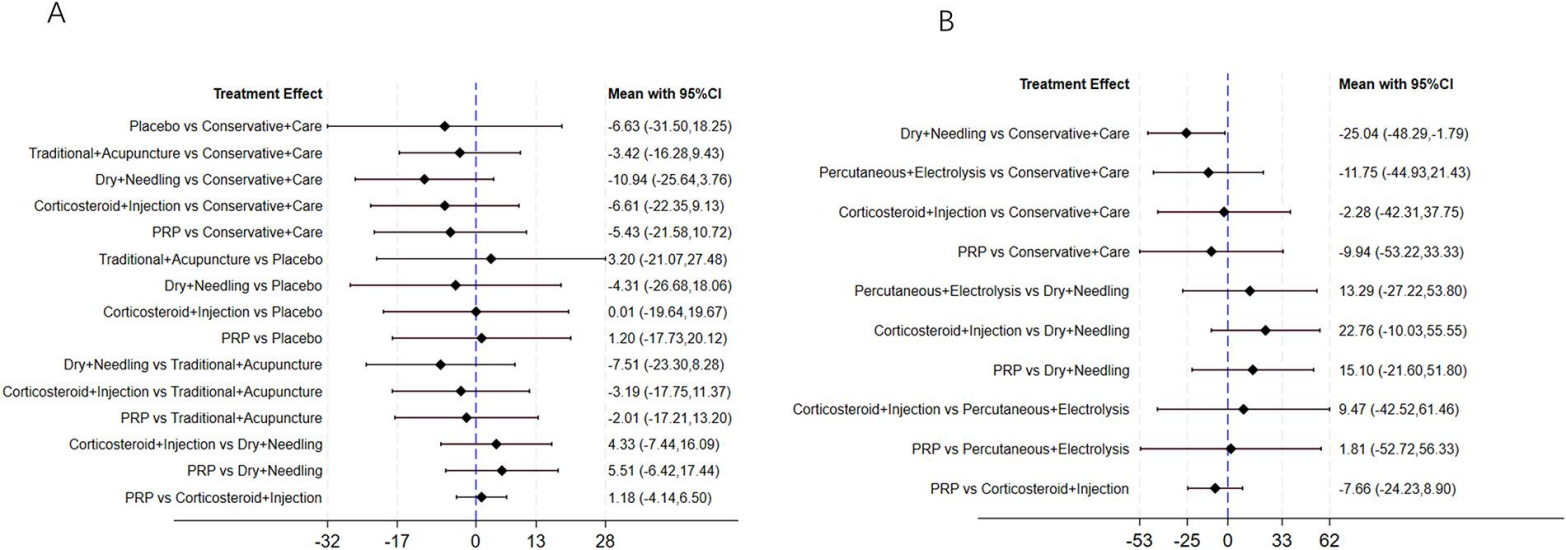
Forest plot of network meta-analysis for DASH and PRTEE comparing all interventions with conservative care [**(A)** DASH, **(B)** PRTEE].

Patient functional outcomes were synthesized through two complementary dimensions (Figures 6, 7, and Table 3): the generalized upper-extremity tool (DASH) and the elbow-specific questionnaire (PRTEE).

**Table 3:** League table for pairwise comparisons of functional recovery (DASH and PRTEE) among different physical interventions for lateral epicondylitis: a network meta-analysis.

Intervention measures	Conservative Care	Placebo	Traditional Acupuncture	Dry Needling	Percutaneous Electrolysis	Corticosteroid Injection	PRP
Conservative Care		−6.63 (−31.50, 18.25)	−3.42 (−16.28, 9.43)	−10.94 (−25.64, 3.76)	—	−6.61 (−22.35, 9.13)	−5.43 (−21.58, 10.72)
Placebo	—		3.20 (−21.0, 27.48)	−4.31 (−26.68, 18.06)	—	0.01 (−19.64, 19.67)	1.20 (−17.73, 20.12)
Traditional Acupuncture	—	—		−7.51 (−23.30, 8.28)	—	−3.19 (−17.75, 11.37)	−2.01 (−17.2, 13.20)
Dry Needling	−25.04 (−48.29, −1.79)*	—	—		—	4.33 (−7.44, 16.09)	5.51 (−6.42, 17.44)
Percutaneous Electrolysis	−11.75 (−44.93, 21.43)	—	—	13.29 (−27.22, 53.80)		—	—
Corticosteroid Injection	−2.28 (−42.31, 37.75)	—	—	22.76 (−10.03, 55.55)	9.47 (−42.52, 61.46)		1.18 (−4.14,6.50)
PRP	−9.94 (−53.22, 33.33)	—	—	15.10 (−21.60, 51.80)	1.81 (−52.72, 56.33)	−7.66 (−24.23, 8.90)	

Data in the upper right triangle represent DASH scores; data in the lower left triangle represent PRTEE scores. Negative MD values indicate superior functional improvement for the respective comparison. Bold values with an asterisk (*) indicate statistical significance. If an indicator is not included in a particular therapy, it is denoted by “—” in the cell.

For the DASH metric: dry needling demonstrated the highest ranking propensity (SUCRA = 79.7%), followed by corticosteroid injections (SUCRA = 57.0%). However, reflecting a lack of true comparative hierarchy, the 95% confidence intervals for all active interventions vs. conservative care heavily spanned the null line (all 95% CIs included 0). This indicated no statistically significant differences between any of the groups under this generalized dimension.

For the PRTEE metric: dry needling maintained a distinct ranking advantage (SUCRA = 85.2%; PrBest = 56.4%). Notably, dry needling emerged as the sole active therapy that achieved a statistically significant improvement over conservative care, yielding a mean difference of −25.04 (95% CI: −48.29, −1.79). When benchmarked against the validated PRTEE MCID threshold (defined as 11 points or a 15% baseline change), the therapeutic effect of dry needling (−25.04) comfortably surpassed this clinical relevance benchmark. Conversely, for the generalized upper-limb functional domain (DASH), where the MCID threshold ranges from 10 to 15 points, none of the active interventions approached or crossed this clinically meaningful boundary relative to conservative care, which structurally aligns with the overlapping SUCRA curves and widespread statistical non-significance observed in this dimension. However, because these regional functional findings are pooled from a relatively restricted sub-network of only 5 primary studies, this therapeutic advantage must be interpreted with caution as a localized trend within a sparse evidence loop.

In summary, a clear clinical bifurcation was observed: while percutaneous electrolysis yielded the highest probability for short-term pain ablation, dry needling exhibited a significant localized advantage for regional functional restoration.

#### 3.4.3 Quantitative heterogeneity and predictive assessment

The quantitative evaluation of the network infrastructure via the global design-inconsistency framework revealed levels of residual variance across the pain and generalized functional dimensions, reflecting the diverse clinical protocols, current intensities, and needle manipulation parameters across the included trials:

For Pain Relief (VAS): The global residual variation due to heterogeneity was flagged as exceptionally high (*I*^2^ = 97.74%, with a between-study variance *τ*² = 3.277). This extensive underlying variance structurally broadens the overall prediction windows of the network. Consequently, while the consistency point estimates validated significant short-term pain ablation for active modalities (most notably percutaneous electrolysis, which achieved a direct coefficient of −6.00 95% CI: −9.75 to −2.25), the wide predictive limits suggest that the magnitude of relief may fluctuate depending on specific clinical settings. This considerable statistical noise empirically supports our conservative decision to downgrade the evidence certainty within the GRADE heterogeneity domain.

**Figure 8:**
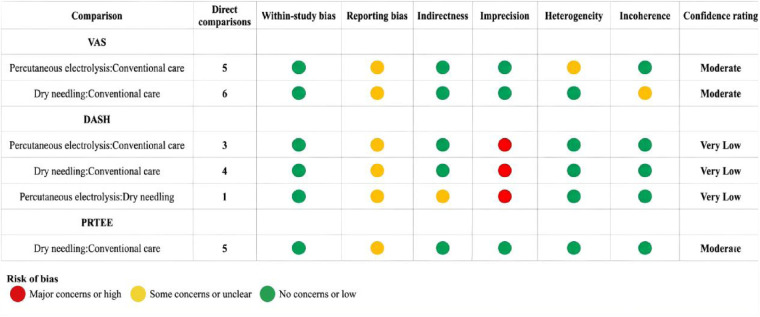
Summary matrix of the GRADE certainty of evidence assessment.

For Generalized Function (DASH): Concurrently, a substantial level of residual variation was observed (*I*^2^ = 96.20%, *τ*² = 77.36). Reflecting this statistical uncertainty, the predictive intervals for all active comparisons—including dry needling and percutaneous electrolysis vs. conventional care—exhibited extreme width and heavily spanned the null value (zero). This pronounced predictive imprecision indicates that clear functional superiority cannot be consistently guaranteed across heterogeneous patient phenotypes, directly aligning with the widespread “Very Low” certainty ratings and “Major Concerns” assigned in our structural GRADE assessment (Figure 8).

In contrast to the closed-loop layouts of the VAS and DASH networks, the PRTEE network presented a strictly acyclic, radial topology centered on conventional care. Because redundant indirect comparative pathways do not mathematically exist, a global residual *I*^2^ model is not statistically applicable. Instead, a local node-splitting analysis was executed to verify the mathematical harmony. This statistical convergence (*P* > 0.05) confirms the stability and transitivity of the PRTEE network within its localized direct comparisons.

### 3.5 GRADE certainty of evidence summary

The comprehensive GRADE certainty-of-evidence profile for key comparisons is illustrated in Figure 8. For the short-term pain relief (VAS outcome), the evidence comparing percutaneous electrolysis or dry needling against conventional care was rated as Moderate, downgraded primarily due to non-significance in prospective clinical registrations (reporting bias) and moderate heterogeneity. In contrast, the evidence for functional outcomes evaluated via the DASH scale was universally graded as Very Low. This pervasive downgrading was driven by severe imprecision (95% CIs cross the null line) and structural indirectness within the network loops. For elbow-specific function (PRTEE), dry needling demonstrated Moderate certainty of evidence compared to conventional care.

### 3.6 Cluster analysis results

**Figure 9:**
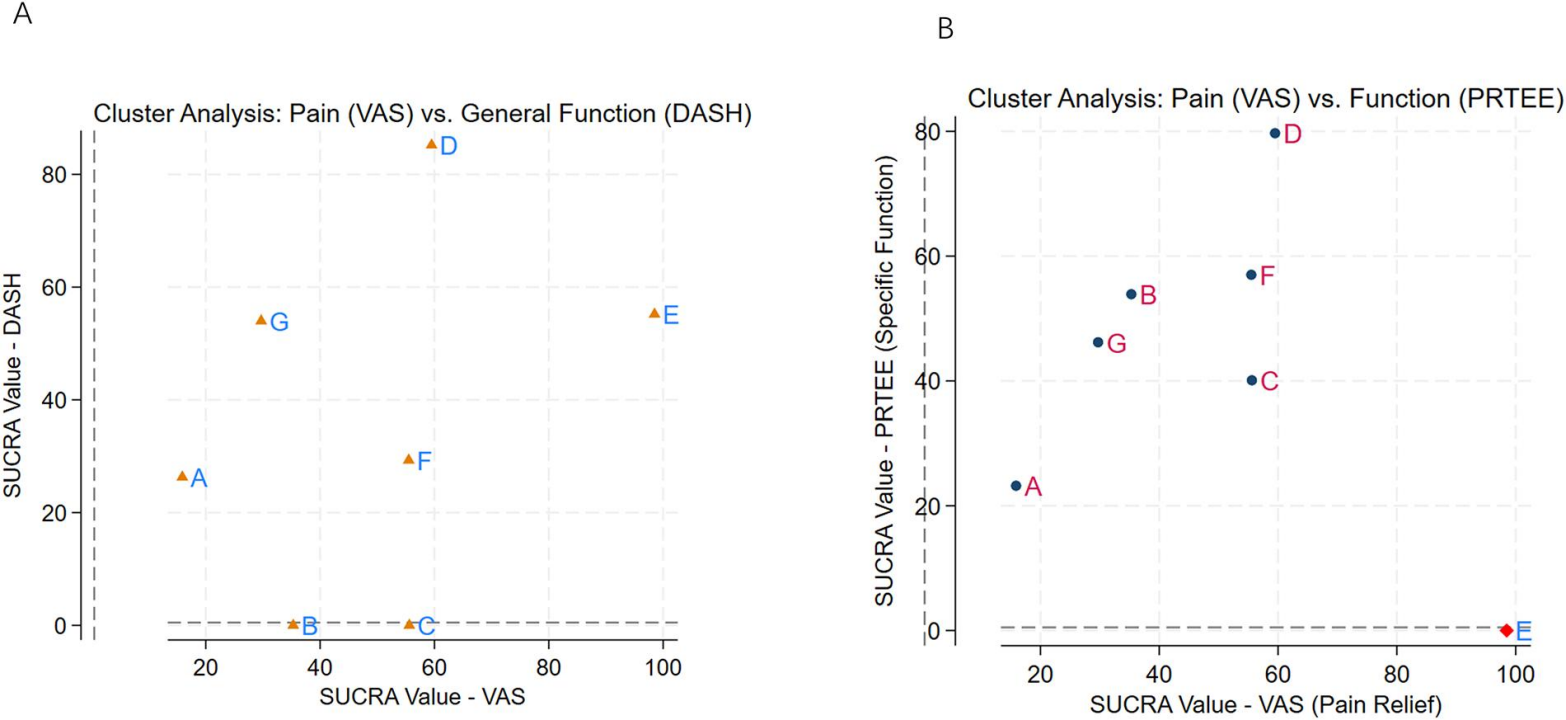
Cluster analysis plot [**(A)** VAS vs DASH, **(B)** VAS vs PRTEE].

To assess the relative advantages of different interventions in pain alleviation and functional restoration, this study conducted a cluster analysis of the SUCRA values associated with each outcome measure. The clustering analysis applied to SUCRA values for the VAS and DASH scores produced the results depicted in Figure 9A. Notably, Percutaneous Electrolysis was positioned at the far right of the scatter plot, signifying its higher probabilistic ranking in pain relief while concurrently achieving substantial improvements in DASH functional scores. This positioning suggests that Percutaneous Electrolysis is the preferred intervention for achieving rapid analgesia. The application of dry needling at the superior aspect of the vertical axis demonstrate optimal potential for the enhancement of overall upper limb function, whilst concurrently exhibiting balanced pain relief capabilities. Corticosteroid injections and PRP were concentrated in the central region of the diagram, indicating comparable performance in terms of pain relief and functional recovery. The findings indicate that both basic treatment and traditional acupuncture were distributed in the lower-left quadrant, suggesting that the overall benefits across both dimensions were relatively limited.

The cluster analysis, which integrated the VAS with elbow-specific PRTEE metrics, demonstrated that dry needling consistently occupied the top position on the vertical axis, thereby reinforcing its role in facilitating elbow-specific functional recovery (Figure 9B). Traditional acupuncture showed enhanced performance in elbow-specific assessments compared to its DASH outcomes, suggesting its greater efficacy in addressing localized elbow symptoms rather than improving overall function. Placebo-controlled conservative treatment remained positioned within the low-efficacy zone of the cluster diagram.

Based on the results of the network meta-analysis and cluster analysis, percutaneous electrolysis demonstrated the most significant efficacy in alleviating tennis elbow pain, exhibiting statistical superiority within this specific framework. Dry needling showed the highest probability advantage in improving patients' overall function and elbow-specific function. Clinicians are advised to tailor treatment selection based on patients' primary clinical concerns: Percutaneous electrolysis is recommended as an effective option for patients experiencing pain that significantly impacts daily life. For patients primarily presenting with activity limitations and functional impairment, dry needling represents a more appropriate therapeutic choice.

### 3.7 Publication bias

**Figure 10:**
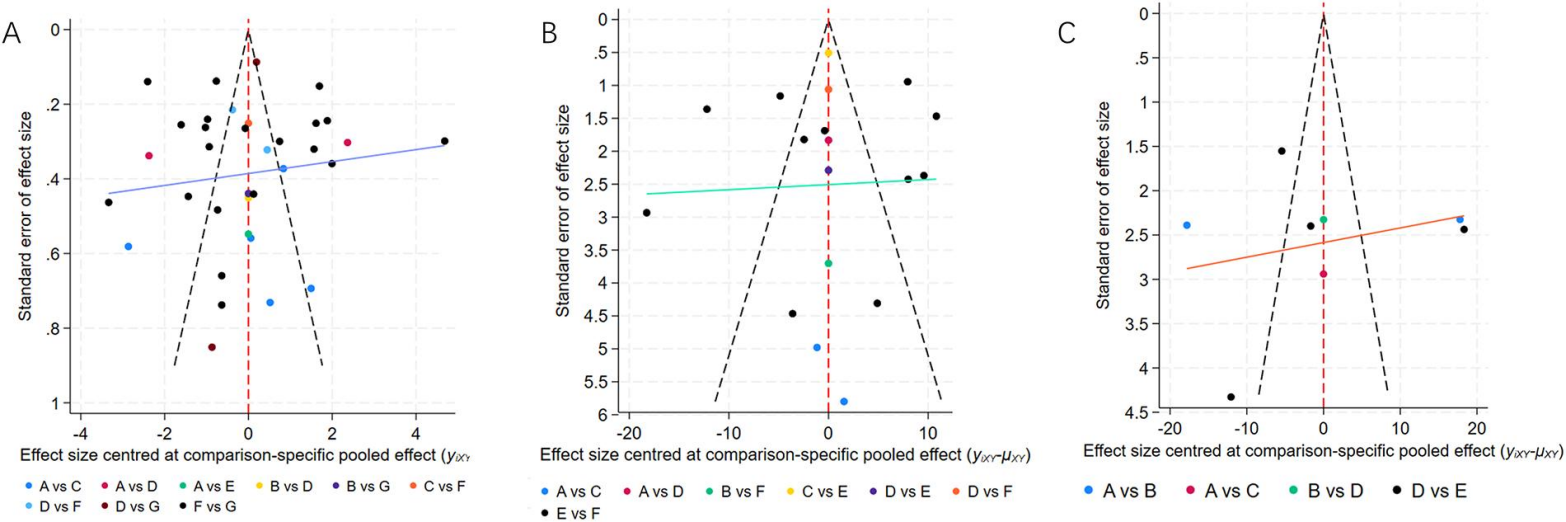
Funnel plot [**(A)** VAS, **(B)** DASH, **(C)** PRTEE].

To scrutinize potential publication bias and systemic small-study effects within the treatment network, comparison-adjusted funnel plots were constructed for the core clinical outcomes (Figures 10A–C). Visual inspection revealed that for the VAS and DASH funnel plots, the trial nodes clustered predominantly symmetrically around the zero-line in the upper and middle tiers of the funnel, indicative of smaller standard errors. The solid lines representing the regression analyses remained largely horizontal or displayed only a minimal slope. This observation suggests that the findings related to pain relief and overall upper limb function are relatively robust against sweeping publication omissions.

Conversely, the funnel plot for the PRTEE indicator demonstrated a sloped regression line and notable asymmetry. Given that the number of studies reporting PRTEE is lower than those for VAS and DASH, this asymmetry is more likely attributable to localized small-study effects rather than pure publication bias. Earlier trials with restricted sample sizes evaluating active micro-invasive modalities (such as dry needling) tended to yield more pronounced point estimates compared to larger-scale replications. Overall, the core outcome measures (VAS and DASH) in this network meta-analysis demonstrated satisfactory stability. Although the PRTEE dimension carries a higher risk of predictive imprecision, the logical consistency in treatment effect trends across all three measures supports the primary conclusions of this study.

## 4 Discussion

This network meta-analysis systematically evaluates the comparative efficacy of various physical and micro-invasive interventions for LE, revealing a distinct “dimensional divergence” in therapeutic outcomes. Our findings demonstrate that PE holds a commanding probabilistic advantage in pain mitigation, ranking highest in SUCRA value (98.5%) within this evidence network. Conversely, DN emerges as the most promising modality for early functional rehabilitation, situational on both the generalized DASH (SUCRA = 79.7%) and elbow-specific PRTEE (SUCRA = 85.2%) hierarchies. Crucially, as captured in our two-dimensional cluster analysis, PE and DN occupy the apexes of the “analgesic efficacy” and “early functional restoration” axes, respectively. This phenomenon highlights a notable therapeutic asynchrony between pain relief and functional recovery in the physical management of LE, underscoring the complexity of LE as a chronic degenerative tendinopathy rather than a transient inflammatory condition.

The core clinical strength of this contemporary evidence synthesis is the cross-validation of statistical pooling against established MCID thresholds. In patients suffering from LE, managing localized acute tendon pain and reclaiming early joint mobility are the priorities. Our NMA demonstrates that within a short-term observational window (≤3 months), PE achieves a VAS reduction of 6.00 points (95% CI: −9.75 to −2.25), which represents a substantial reduction exceeding the conservative MCID barrier (1.5–2.0 points). Similarly, the functional utility of DN on the PRTEE scale −25.04 points (95% CI: −48.29 to −1.79). clinically exceeded its corresponding MCID benchmark (11.0 points), validating its role as a robust tool for localized structural restoration. However, the failure of all physical modalities to breach the MCID threshold on the DASH scale is highly telling. Because the DASH is a generalized upper-limb instrument encompassing holistic activities, localized micro-invasive therapies onto the extensor tendon may not immediately manifest as sweeping changes in macro-functional daily living scores within a short-term evaluation phase, an insight that clinicians must weigh carefully during shared decision-making.

In alignment with the angiofibroblastic hyperplasia theory, the core pathology of LE involves dysfunctional tendon remodeling and chronic degenerative changes induced by mechanical overloading (8–11),The clinical superiority of PE in VAS metrics is intrinsically linked to its unique local electrochemical effects, moving beyond traditional conservative protocols or anti-inflammatory drugs that may inadvertently interfere with the healing process by inhibiting tenocyte activity (12–15). Administered under ultrasound guidance to precisely target the pathological tendon matrix (16, 17). PE delivers a galvanic current via a cathode needle to trigger a localized electrochemical reaction, producing electrolytic products such as sodium hydroxide (NaOH) (18, 19). This rapid alkalization of the microenvironment induces a controlled “chemical ablation” that shifts the biological state from chronic degeneration to acute repair, effectively breaking the nociceptive cycle. Furthermore, PE more effectively modulates regional pH levels, activates the NLRP3 inflammasome to initiate fibroblast proliferation, and drives segmental neuromodulation via high-intensity currents, thereby achieving rapid and immediate analgesic effects (20–22).

Distinct from the electrochemical paradigm of PE, DN utilizes non-pharmacological filiform needles grounded in Western anatomical principles to modulate neuromusculoskeletal nociception and remediate motor dysfunction (23). In the management of LE, DN targeting strategies focus on the common extensor origin and the muscle belly of the forearm extensors to optimize the neuromuscular kinetic chain. Evidence suggests that needle-induced mechanical micro-trauma can downregulate biomarkers such as substance *P* and calcitonin gene-related peptide, alter cholinergic activity, and elevate *β*-endorphin levels, contributing to peripheral desensitization (24–26).

The core objective of DN within the muscle belly is the inactivation of myofascial trigger points (MTrPs), which are defined as hypersensitive focal zones within taut bands of skeletal muscle (27–29). By eliminating these spasmodic taut bands, DN directly restores the muscle's length-tension relationship, providing superior mechanical support during functional tasks such as gripping or rotation. Moreover, the mechanical stimulation induces localized axon reflexes, enhancing regional blood flow and fibroblast chemotaxis to accelerate extracellular matrix remodeling (30, 31), which explains its favorable performance in improving DASH and PRTEE scores within sub-acute observational windows (32).

The cluster analysis in this study provides intuitive evidence for personalized clinical decision-making based on patient phenotyping. For patients in the acute phase presenting with high pain sensitivity, PE should be prioritized to achieve rapid symptomatic relief and enhance initial treatment adherence. Conversely, for those in the chronic or sub-acute phases characterized by diminished grip strength or significant functional impairment in daily activities, DN serves as the core intervention for achieving high-quality functional rehabilitation. Regarding PRP and corticosteroid injections, despite their widespread traditional use, their comprehensive ranking within this evidence network remains moderate, and their clinical superiority over conventional care failed to reach statistical significance in the functional recovery dimension, reinforcing the emerging shift toward needle-based physical modalities.

To ensure the validity of our NMA findings, the transitivity assumption was carefully examined. Clinically, lateral epicondylitis (LE) presentations can vary based on symptom duration (acute vs. chronic) and baseline severity. In our review, the majority of the included RCTs predominantly evaluated patients with chronic LE (symptom duration > 3 months) and moderate-to-severe baseline pain, which conceptually underpins a homogeneous clinical framework. Methodologically, although variations in intervention protocols exist (such as the intensity of percutaneous electrolysis or the specific needling depth in dry needling), all treatments were integrated under conceptual categories with tightly matching short-term follow-up windows (1–3 months). Concomitant treatments were largely restricted to standard eccentric exercises or educational advice across arms, minimizing the confounding effects of overlapping therapies. Therefore, the transitivity assumption holds reasonably well, lending high transparency and credibility to the compiled comparative hierarchy.

The subtle topological asymmetry captured in our comparison-adjusted funnel plot warrants critical methodological reflection regarding small-study effects in micro-invasive physical therapy literature. In network meta-analyses, a skewed funnel distribution is rarely driven by a singular source. While selective publication remains a systemic risk, the observed lower-tail scattering in our study is heavily characteristic of small-scale trials. Small-scale trials often lack prospective clinical registration or suffer from inadequate blinding protocols during aggressive needle-based procedures, structurally inflating the pooled effect sizes in early evidence loops. This empirical vulnerability heavily corroborates the rationale behind our conservative GRADE certainty downgrading, specifically within the reporting bias and imprecision domains for the DASH and VAS networks.

Importantly, the high global residual heterogeneity captured in our random-effects models (VAS: *I*^2^ = 97.74%; DASH: *I*^2^ = 96.20%) serves as a crucial quantitative boundary for our findings. In contemporary sports medicine, drawing definitive or far-reaching long-term clinical recommendations from an empirical database derived predominantly from short-term trials (≤3 months) is an interpretive risk. Acknowledging the temporal boundaries and high statistical variance of the available evidence is paramount to preventing clinical over-interpretation. Therefore, we do not recommend formulating definitive long-term clinical guidelines based on these early SUCRA ranking tendencies. Clinicians must interpret these magnitudes with realistic boundary expectations, balancing immediate, early symptom relief with the lack of longitudinal follow-up data (6–12 months) regarding permanence and recurrence rates.

## 5 Limitation

Several inherent methodological and clinical limitations should be considered when interpreting the findings of this network meta-analysis.

First, a pronounced level of residual statistical heterogeneity was observed within our global random-effects models, particularly for the pain (VAS: *I^2^* = 97.74%, *τ*² = 3.277) and generalized functional networks (DASH: *I^2^* = 96.20%, *τ*² = 77.36). In contemporary sports medicine and micro-invasive rehabilitation literature, such substantial variance represents a common and predictable reality, driven by unstandardized, technician-dependent operational variables across different global trial sites. Crucially, rather than masking this variance, we addressed this limitation with strict academic transparency by utilizing these high *I*^2^ and *τ*²parameters as the empirical foundation to systematically penalize our certainty-of-evidence framework. Consequently, within our adapted GRADE assessment, metrics displaying extensive predictive imprecision were rigorously downgraded to “Very Low” certainty, establishing a conservative and legally safe boundary for clinical translation.

Second, there is a notable disparity in the volume and structural density of empirical evidence across different outcome measures. While the closed-loop networks for VAS and DASH scores were relatively well-saturated, the evidence layout for elbow-specific function (PRTEE) was comprised of a restricted sub-network of only 5 original trials arranged in a strictly acyclic, radial topology. Because independent indirect comparative pathways do not mathematically exist within this tree-like configuration, a global residual heterogeneity model could not be computed. Although a rigorous local node-splitting analysis (network sidesplit all) indicated statistical consistency (*P* > 0.05), the sheer sparsity of this localized sub-network naturally increases its vulnerability to potential small-study effects or replication variance. Recognizing this structural constraint, we intentionally capped the GRADE certainty of the “Dry needling vs. Conventional care” PRTEE loop at a “Moderate” level rather than “High”, and clinicians should weigh its observed superiority against the limited baseline sample scales.

Third, while the SUCRA rankings and cluster analyses provide an informative hierarchy of physical interventions, they remain strictly probabilistic tools. Because several direct and indirect pairwise comparisons did not reach statistical significance and displayed wide confidence intervals, the precise ranking curves reflect statistical mathematical propensities rather than definitive, unshakeable clinical superiority when head-to-head dominance is absent.

Fourth, the macro-level classification utilized in this study inevitably glosses over the fine-grained therapeutic nuances within certain treatment arms, most notably the dry needling and conservative care protocols. Our analysis aggregates deep, target-specific trigger-point needling with more superficial or generalized structural needling under a single unified node. These technical variations—encompassing differences in needle gauge, retention times, and manipulation frequencies—naturally fed into the unexplained clinical heterogeneity observed within our functional and pain outcomes. Future research utilizing larger, standardized multi-center cohorts should aim to perform component-resolved network meta-analyses to dissect the independent efficacy profiles of these technical subtypes.

Finally, potential clinical heterogeneity arises from variations in individual patient baselines and chronological dimensions. Parameters such as individual symptom duration and prior injection history across trial cohorts could not be entirely controlled, posing theoretical challenges to transitivity. Furthermore, the current empirical database is predominantly restricted to short-term observational windows, typically ranging from 1 to 3 months post-intervention. Given the chronic and relapsing pathophysiology of lateral epicondylitis, the complete absence of longitudinal follow-up data (e.g., 6–12 months) limits our ability to gauge the permanence, recurrence rates, or long-term safety margins of these micro-invasive modalities. Therefore, we do not recommend formulating definitive long-term clinical guidelines based on these early temporal profiles until verified by extended-window randomized controlled trials.

## 6 Conclusion

In summary, our network meta-analysis demonstrates that within a short-term observational window (≤3 months),percutaneous electrolysis holds the highest probabilistic propensity for rapid pain ablation, whereas dry needling exhibits a favorable probabilistic propensity for early regional functional restoration in patients with chronic lateral epicondylitis. However, given that the differences in DASH scores did not reach statistical significance, its more appropriately framed framed as a potential clinical trend rather than established superiority. Given that the underlying empirical evidence is predominantly restricted to short-term follow-ups, these hierarchical ranking tendencies must be translated into clinical practice with appropriate caution. We do not recommend formulating definitive long-term clinical guidelines based on these early temporal profiles. Future high-quality, large-sample randomized trials with longitudinal follow-up synchronized at 6 to 12 months remain mandatory to verify the long-term sustainability, recurrence rates, and comparative safety margins of these micro-invasive modalities.

## Data Availability

The original contributions presented in the study are included in the article/Supplementary Material, further inquiries can be directed to the corresponding author.

## References

[1] Keijsers R de Vos RJ Kuijer PPFM van den Bekerom MPJ van der Woude HJ Eygendaal D. Tennis elbow. Shoulder Elbow. (2019) 11:384–92. 10.1177/1758573218797973 31534489 PMC6739751

[2] Sayadi S Shahbazi P Najafi A Ochi F Jafarabady K Rezaei MM. Platelet-rich plasma versus corticosteroid: a randomized controlled trial on tennis elbow patients resistant to nonsurgical treatments. Ann Med Surg. (2023) 85:4385–8. 10.1097/MS9.0000000000001115 PMC10473312 37663722

[3] Xu Y Li T Wang L Yao L Li J Tang X. Platelet-rich plasma has better results for long-term functional improvement and pain relief for lateral epicondylitis: a systematic review and meta-analysis of randomized controlled trials. Am J Sports Med. (2024) 52:2646–56. 10.1177/03635465231213087 38357713

[4] Goyal T Choudhury AK Paul S Sethy SS Singh V Yadav RK. Outcomes of continued intensive conservative treatment versus arthroscopic extensor carpi radialis brevis release for recalcitrant lateral epicondylitis: a non-randomized controlled trial. Indian J Orthop. (2022) 56:1578–86. 10.1007/s43465-022-00649-w 36052381 PMC9385926

[5] Maroun R Daher M Boufadel P Lopez R Khan AZ Abboud JA. Platelet rich plasma versus corticosteroids for lateral epicondylitis: a meta-analysis of randomized clinical trials. Clin Shoulder Elb. (2025) 28:40–8. 10.5397/cise.2024.00801 40077872 PMC11938920

[6] Bonczar M Ostrowski P Plutecki D Dziedzic M Florek J Michalik W. Treatment options for tennis elbow—an umbrella review. Folia Med Cracov. (2023) 63:31–58. 10.24425/fmc.2023.147213 38310528

[7] Pathan AF Sharath HV. A review of physiotherapy techniques used in the treatment of tennis elbow. Cureus. (2023) 15:e47706. 10.7759/cureus.47706 38021828 PMC10674892

[8] Loiacono C Palermi S Massa B Belviso I Romano V Di Gregorio A. Tendinopathy: pathophysiology, therapeutic options, and role of nutraceutics. A narrative literature review. Medicina (B Aires). (2019) 55:447. 10.3390/medicina55080447 PMC6723894 31394838

[9] Girgis B Duarte JA. Physical therapy for tendinopathy: an umbrella review of systematic reviews and meta-analyses. Phys Ther Sport. (2020) 46:30–46. 10.1016/j.ptsp.2020.08.002 32877858

[10] Millar NL Silbernagel KG Thorborg K Kirwan PD Galatz LM Abrams GD. Tendinopathy. Nat Rev Dis Primers. (2021) 7:1. 10.1038/s41572-020-00234-1 33414454

[11] Clifford C Challoumas D Paul L Syme G Millar NL. Effectiveness of isometric exercise in the management of tendinopathy: a systematic review and meta-analysis of randomised trials. Bmj Open Sport Exerc Med. (2020) 6:e760. 10.1136/bmjsem-2020-000760 PMC7406028 32818059

[12] Challoumas D Clifford C Kirwan P Millar NL. How does surgery compare to sham surgery or physiotherapy as a treatment for tendinopathy? A systematic review of randomised trials. Bmj Open Sport Exerc Med. (2019) 5:e528. 10.1136/bmjsem-2019-000528 PMC6539146 31191975

[13] Day JM Lucado AM Uhl TL. A comprehensive rehabilitation program for treating lateral elbow tendinopathy. Int J Sports Phys Ther. (2019) 14:818–29. 10.26603/ijspt20190818 31598419 PMC6769266

[14] Irby A Gutierrez J Chamberlin C Thomas SJ Rosen AB. Clinical management of tendinopathy: a systematic review of systematic reviews evaluating the effectiveness of tendinopathy treatments. Scand J Med Sci Sports. (2020) 30:1810–26. 10.1111/sms.13734 32484976

[15] Rees JD Maffulli N Cook J. Management of tendinopathy. Am J Sports Med. (2009) 37:1855–67. 10.1177/0363546508324283 19188560

[16] Al-Boloushi Z Gómez-Trullén EM Arian M Fernández D Herrero P Bellosta-López P. Comparing two dry needling interventions for plantar heel pain: a randomised controlled trial. Bmj Open. (2020) 10:e38033. 10.1136/bmjopen-2020-038033 PMC7440826 32819949

[17] Rodríguez-Huguet M Góngora-Rodríguez J Rodríguez-Huguet P Ibañez-Vera AJ Rodríguez-Almagro D Martín-Valero R. Effectiveness of percutaneous electrolysis in supraspinatus tendinopathy: a single-blinded randomized controlled trial. J Clin Med. (2020) 9:1837. 10.3390/jcm9061837 32545583 PMC7356532

[18] Ferreira MHL Araujo GAS De-La-Cruz-Torres B. Effectiveness of percutaneous needle electrolysis to reduce pain in tendinopathies: a systematic review with meta-analysis. J Sport Rehabil. (2024) 33:307–16. 10.1123/jsr.2024-0009 38897578

[19] Yildizgoren MT Bagcier F. A modern interpretation of traditional galvanic current: percutaneous needle electrolysis therapy. Acupunct Med. (2024) 42:56–7. 10.1177/09645284231210569 38014666

[20] Peñin-Franch A García-Vidal JA Martínez CM Escolar-Reina P Martínez-Ojeda RM Gómez AI. Galvanic current activates the nlrp3 inflammasome to promote type i collagen production in tendon. Elife. (2022) 11:e73675. 10.7554/eLife.73675 35199642 PMC8896827

[21] Sánchez-Sánchez JL Calderón-Díez L Herrero-Turrión J Méndez-Sánchez R Arias-Buría JL Fernández-De-Las-Peñas C. Changes in gene expression associated with collagen regeneration and remodeling of extracellular matrix after percutaneous electrolysis on collagenase-induced achilles tendinopathy in an experimental animal model: a pilot study. J Clin Med. (2020) 9:3316. 10.3390/jcm9103316 33076550 PMC7602800

[22] Peñin-Franch A García-Vidal JA Gómez AI Escolar-Reina P Medina-Mirapeix F Pelegrín P. The total electric charge and time of application of galvanic currents to macrophages can optimize the release of il-1β with low cell death. Sci Rep. (2024) 14:30871. 10.1038/s41598-024-81848-3 39730677 PMC11681040

[23] Dunning J Butts R Mourad F Young I Flannagan S Perreault T. Dry needling: a literature review with implications for clinical practice guidelines. Phys Ther Rev. (2014) 19:252–65. 10.1179/108331913X13844245102034 25143704 PMC4117383

[24] Shah JP Danoff JV Desai MJ Parikh S Nakamura LY Phillips TM. Biochemicals associated with pain and inflammation are elevated in sites near to and remote from active myofascial trigger points. Arch Phys Med Rehabil. (2008) 89:16–23. 10.1016/j.apmr.2007.10.018 18164325

[25] Shah JP Phillips TM Danoff JV Gerber LH. An *in vivo* microanalytical technique for measuring the local biochemical milieu of human skeletal muscle. J Appl Physiol. (2005) 99:1977–84. 10.1152/japplphysiol.00419.2005 16037403

[26] Hsieh YL Yang CC Liu SY Chou LW Hong CZ. Remote dose-dependent effects of dry needling at distant myofascial trigger spots of rabbit skeletal muscles on reduction of substance p levels of proximal muscle and spinal cords. Biomed Res Int. (2014) 2014:982121. 10.1155/2014/982121 25276839 PMC4168154

[27] Vázquez-Delgado E Cascos-Romero J Gay-Escoda C. Myofascial pain syndrome associated with trigger points: a literature review. (I): epidemiology, clinical treatment and etiopathogeny. Med Oral Patol Oral Cir Bucal. (2009) 14:e494–8. 10.4317/medoral.14.e494 19680218

[28] Sánchez Romero EA Fernández Carnero J Villafañe JH Calvo-Lobo C Ochoa Sáez V Burgos Caballero V. Prevalence of myofascial trigger points in patients with mild to moderate painful knee osteoarthritis: a secondary analysis. J Clin Med. (2020) 9:2561. 10.3390/jcm9082561 32784592 PMC7464556

[29] Bağcıer F Yurdakul OV. Attention to soft tissues in hip pain: the importance of myofascial trigger point of the iliopsoas muscle in hip osteoarthritis. Agri. (2023) 35:183–4. 10.14744/agri.2021.54837 37493481

[30] González-Iglesias J Cleland JA Del Rosario Gutierrez-Vega M Fernández-De-Las-Peñas C. Multimodal management of lateral epicondylalgia in rock climbers: a prospective case series. J Manipulative Physiol Ther. (2011) 34:635–42. 10.1016/j.jmpt.2011.09.003 22018577

[31] Ma X Qiao Y Wang J Xu A Rong J. Therapeutic effects of dry needling on lateral epicondylitis: an updated systematic review and meta-analysis. Arch Phys Med Rehabil. (2024) 105:2184–97. 10.1016/j.apmr.2024.02.713 38484834

[32] Rabanal-Rodríguez G Navarro-Santana MJ Valera-Calero JA Gómez-Chiguano GF Kocot-Kępska M Fernández-De-Las-Peñas C. Neurophysiological effects of dry needling: a systematic review and meta-analysis. Arch Phys Med Rehabil. (2026) 107:299–314. 10.1016/j.apmr.2025.08.019 40921318

